# A Diverging Species within the *Stewartia gemmata* (Theaceae) Complex Revealed by RAD-Seq Data

**DOI:** 10.3390/plants13101296

**Published:** 2024-05-08

**Authors:** Hanyang Lin, Wenhao Li, Yunpeng Zhao

**Affiliations:** 1 Zhejiang Provincial Key Laboratory of Plant Evolutionary Ecology and Conservation, School of Life Sciences, Taizhou University, Taizhou 318000, China; hylin@tzc.edu.cn; 2 Systematic & Evolutionary Botany and Biodiversity Group, MOE Key Laboratory of Biosystems Homeostasis & Protection, College of Life Sciences, Zhejiang University, Hangzhou 310058, China; wenhao_li@zju.edu.cn

**Keywords:** nascent species, peripatric distribution, RAD-seq, phylogenomic analysis, taxonomic implication

## Abstract

Informed species delimitation is crucial in diverse biological fields; however, it can be problematic for species complexes. Showing a peripatric distribution pattern, *Stewartia gemmata* and *S. acutisepala* (the *S. gemmata* complex) provide us with an opportunity to study species boundaries among taxa undergoing nascent speciation. Here, we generated genomic data from representative individuals across the natural distribution ranges of the *S. gemmata* complex using restriction site-associated DNA sequencing (RAD-seq). Based on the DNA sequence of assembled loci containing 41,436 single-nucleotide polymorphisms (SNPs) and invariant sites, the phylogenetic analysis suggested strong monophyly of both the *S. gemmata* complex and *S. acutisepala*, and the latter was nested within the former. Among *S. gemmata* individuals, the one sampled from Mt. Tianmu (Zhejiang) showed the closest evolutionary affinity with *S. acutisepala* (which is endemic to southern Zhejiang). Estimated from 2996 high-quality SNPs, the genetic divergence between *S. gemmata* and *S. acutisepala* was relatively low (an *F*_st_ of 0.073 on a per-site basis). Nevertheless, we observed a proportion of genomic regions showing relatively high genetic differentiation on a windowed basis. Up to 1037 genomic bins showed an *F*_st_ value greater than 0.25, accounting for 8.31% of the total. After SNPs subject to linkage disequilibrium were pruned, the principal component analysis (PCA) showed that *S. acutisepala* diverged from *S. gemmata* along the first and the second PCs to some extent. By applying phylogenomic analysis, the present study determines that *S. acutisepala* is a variety of *S. gemmata* and is diverging from *S. gemmata*, providing empirical insights into the nascent speciation within a species complex.

## 1. Introduction

Species are the fundamental units of biodiversity, yet the ways in which species have been defined have been a longstanding topic since the epoch of Charles Darwin [1]. Historically, various species concepts and criteria for defining species have been proposed and discussed [2,3,4,5,6,7,8,9], and a general agreement has been reached that species are lineages in terms of biological, evolutionary, ecological, or phenetic aspects, in either a broad or strict sense [10]. Therefore, informed species delimitation should be based on integrated evidence [11,12]. If speciation events occurred a long time ago, species may well fit certain species concepts with strong evidence; however, issues can become complicated when species are still at the nascent stage (i.e., speciation is not complete) [13]. Some examples are as follows: (1) lineages diversify with indistinguishable morphological changes, also known as ‘cryptic/sibling species’ [14] or ‘non-adaptive radiation’ [15]; (2) the presence of discernible phenotypic divergence but little genetic differentiation, which is not rare in adaptive radiations where lineage boundaries are porous [16,17,18]; (3) lineages with morphological and genetic distinctions arise from a former metapopulation, i.e., the case of progenitor-derivative relationships, in which paraphyletic assemblages of populations are predicted to be common [10]. The practice of species delimitation within species complexes that fall into the abovementioned situations will be challenging yet important given that it would largely facilitate our understanding of the patterns and causes at the initial step of speciation [19].

Showing a disjunct distribution and a species diversity disparity between eastern Asia and eastern North America (ca. 18 vs. 2 spp.), *Stewartia* L. (an early-diverged lineage in Theaceae) provides us with an ideal system for studying speciation mechanisms and local adaptation, along with its taxonomic difficulties [20,21,22,23,24]. Among Asian *Stewartia* species, *S. sinensis* Rehder & E. H. Wilson was recognized as having the largest distribution area and highest phenotypic diversity [21,22]. According to *Flora of China (FOC)*, *S. sinensis* harbors four varieties, namely *S. sinensis* var. *sinensis*, *S. sinensis* var. *acutisepala* (P. L. Chiu & G. R. Zhong) T. L. Ming & J. Li (=*S. acutisepala* P. L. Chiu & G. R. Zhong), *S. sinensis* var. *brevicalyx* (S. Z. Yan) T. L. Ming & J. Li (=*S. brevicalyx* S. Z. Yan), and *S. sinensis* var. *shensiensis* (Hung T. Chang) T. L. Ming & J. Li [22]. With an integrated taxonomic approach, recent research decoupled *S. sinensis* into two distinct species based on lines of evidence from phylogenetic affinities, climatic niche volumes, and key morphological traits [25]. The geographically northern lineage (including part of *S. sinensis* var. *sinensis* and *S. sinensis* var. *shensiensis* recognized by *FOC*) comprised the species entity of *S. sinensis*, while the geographically southern lineage (including part of *S. sinensis* var. *sinensis*, *S. sinensis* var. *acutisepala*, and *S. sinensis* var. *brevicalyx* recognized by *FOC*) was assigned the name of *S. gemmata* S. S. Chien & W. C. Cheng, a legitimate name with resurrection [22,26]. Despite the clarification of the species boundary between *S. sinensis* and *S. gemmata*, the taxonomic relationships among *S. gemmata*, *S. acutisepala*, and *S. brevicalyx* (the *S. gemmata* complex, hereafter) remain elusive (Table S1). In the biogeographic sense, *S. gemmata* mainly inhabits temperate broadleaf and mixed forests in eastern to southern China (mainly Zhejiang, Anhui, Jiangxi, Fujian, Hunan, Guangxi, and Guangdong), *S. acutisepala* is endemic to mountainous regions in southern Zhejiang of eastern China, and *S. brevicalyx* can only be found at Mt. Tianmu (northern Zhejiang) and its adjacent areas according to the literature [20,25,27]. Therefore, *S. acutisepala* shows a peripatric distribution with *S. gemmata*, and *S. brevicalyx* is sympatric with *S. gemmata* but with extremely limited distribution (if it exists). Morphologically, *S. acutisepala* mainly differs from *S. gemmata* in the exfoliation pattern of barks, while no evident differences were found between *S. gemmata* and *S. brevicalyx*, as documented in a recently revised regional flora [28]. A molecular dating analysis suggested that *S. acutisepala* diverged from *S. gemmata* ca. 2 million years ago as its sister group, though the robustness of this inference remains to be tested given that only a few individuals were included [24]. Therefore, we hypothesize that *S. acutisepala* is a variety of *S. gemmata* or a sister species of *S. gemmata*, and *S. brevicalyx* is likely conspecific with *S. gemmata*. In summary, the *S. gemmata* complex (especially *S. gemmata* and *S. acutisepala*) is a good candidate for exploring the existence of nascent species and the underlying speciation mechanisms, and a deeper investigation into species boundaries within this species complex is called for.

Taking advantage of the rapid development of DNA sequencing technologies, systematists are now embracing nuclear genomic data to decipher the evolutionary history of diverse organisms [29]. Specifically, an increasing body of studies has harnessed the power of single-nucleotide polymorphisms (SNPs) to resolve recalcitrant phylogenetic relationships [30]. Compared to alternative genomic markers, SNPs are abundant genome-wide and easy to collect. Emerging techniques for the de novo sequencing and identification of SNPs, such as restriction site-associated DNA sequencing (RAD-seq), are increasingly welcomed in evolutionary research [30,31,32,33]. By discovering and screening SNPs in a fraction of the genome, RAD-seq could generate massive SNPs with relatively low economic and time costs, which have been proven efficient in empirical species delimitation studies [34,35,36,37,38,39]. Therefore, utilizing RAD-seq data to trace the evolutionary imprints of the *S. gemmata* complex is promising.

To test whether *S. acutisepala* is a variety or a sister species of *S. gemmata*, we sampled representative individuals of the *S. gemmata* complex across its native ranges and generated genomic data using RAD-seq to ask if the two species represent distinct evolutionary lineages, and if so, how divergent they are from one another.

## 2. Results

### 2.1. Assembled Data

After filtering the adapter sequences and low-quality reads, the RAD-seq generated an average of 3.75 Gb (2.75–5.45 Gb) of clean data for each sample (Table 1). With its default settings, the ipyrad pipeline retained a total of 41,436 SNPs, of which each was shared by at least four individuals. The sequence alignment of assembled loci (including invariant sites) was 1,808,596 bp long. A detailed statistical summary of assembled loci among 11 accessions is available in Table S2.

### 2.2. Phylogenetic Relationship of S. gemmata and S. acutisepala

**Figure 1:**
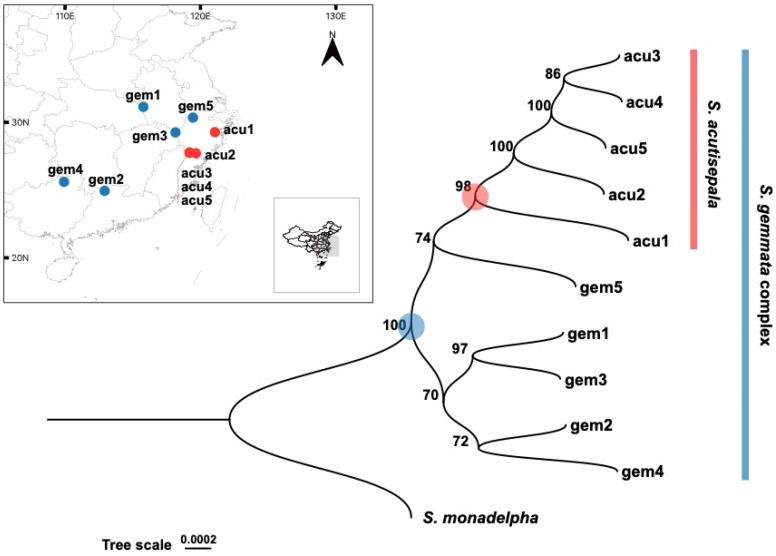
Geographic distribution of studied individuals of *Stewartia gemmata* complex and the phylogenetic tree showing the evolutionary affinities among these plants. The distribution ranges (shadowed) of *S. gemmata* and *S. acutisepala* were drawn based on [22,25,28]. In the consensus maximum likelihood (ML) tree, ‘gem’ stands for *S. gemmata* (blue) and ‘acu’ stands for *S. acutisepala* (red). The bootstrap support value is shown for each node. The scale bar is in the unit of the number of substitutions per site. The map was retrieved from the Geospatial Data Cloud, Computer Network Information Center, Chinese Academy of Sciences (http://www.gscloud.cn/; accessed on 12 November 2023). The accession IDs are consistent with those in Table 1.

Among 484 tested models of DNA substitution, TPM3u + F + I was determined to be the best-fit model of the sequence alignment according to the Bayesian Information Criterion (4,984,151.533). The consensus maximum likelihood (ML) phylogenetic tree (log-likelihood = −2,491,895.601; total tree length = 0.013) suggested a strong monophyly of the *S. gemmata* complex (bootstrap support (BS) = 100; Figure 1).

Within the *S. gemmata* complex, four *S. gemmata* individuals formed a clade (BS = 70). Among them, gem1 (sampled from Mt. Tiantangzhai, Anhui) was sister to gem3 (sampled from Mt. Gutian, Zhejiang) (BS = 97), and gem2 (sampled from Mt. Mang, Hunan) showed a sister relationship with gem4 (sampled from Huaping National Nature Reserve, Guangxi) (BS = 72). This clade was sister to another clade (BS = 74) consisting of one *S. gemmata* individual (gem5; sampled from Mt. Tianmu, Zhejiang) and all *S. acutisepala* individuals (Figure 1).

The five studied *S. acutisepala* individuals formed a strongly supported clade (BS = 98), which was nested within other *S. gemmata* individuals (Figure 1). Within the *S. acutisepala* clade, acu1 (sampled from Mt. Tiantai) and acu2 (sampled from Wuyanling National Nature Reserve) diverged sequentially, and the three accessions from Mt. Baishanzu (acu3, acu4, and acu5), showed close affinities (BS = 100).

### 2.3. Genetic Differentiation within the S. gemmata Complex

**Figure 2:**
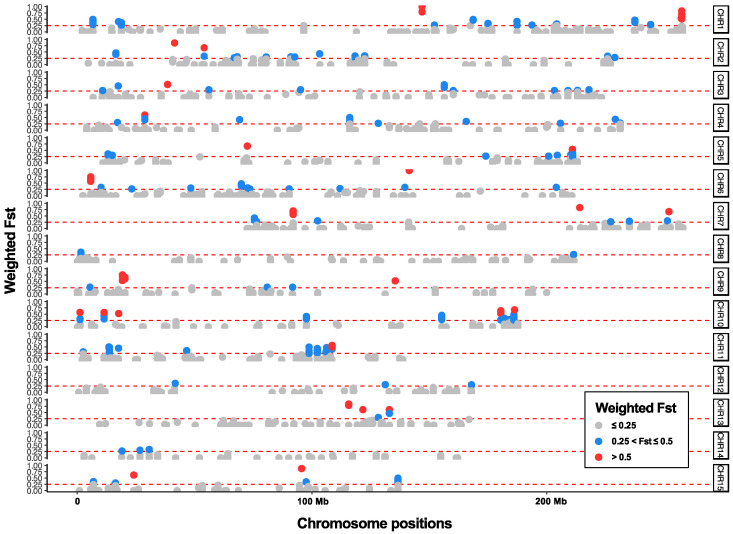
Genetic differentiation between *S. gemmata* and *S. acutisepala* measured using weighted Weir and Cockerham’s *F*_st_ on a windowed basis. The dashed line indicates an *F*_st_ value greater than 0.25 in each pseudo-chromosome. The red, blue, and gray genomic bins indicate *F*_st_ values > 0.50, 0.50–0.25, and ≤0.25, respectively.

The filtering procedure resulted in a dataset containing 2996 high-quality SNPs. Based on these SNPs, the overall weighted Weir and Cockerham’s *F*_st_ between *S. gemmata* and *S. acutisepala* was estimated at 0.073 on a per-site basis. Among 12,482 genomic bins, 11,445 bins (91.69%) showed an *F*_st_ value equal to or less than 0.25, while 1037 bins showed an *F*_st_ value greater than 0.25 (8.31%). More specifically, 787 bins (6.31%) showed an *F*_st_ value greater than 0.25 but equal to or less than 0.5, and 250 bins (2.00%) showed an *F*_st_ value greater than 0.5 (Figure 2).

**Figure 3:**
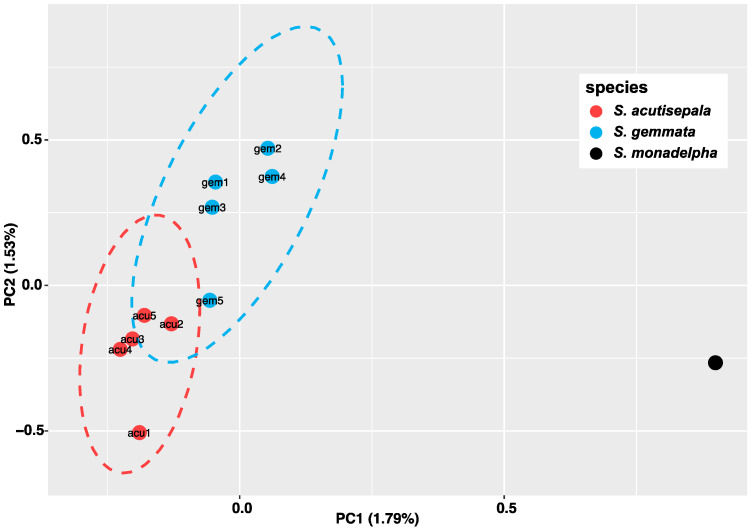
The distribution of five *S. gemmata* individuals (blue dots), five *S. acutisepala* individuals (red dots), and one *S. monadelpha* individual (the black dot) along the first and the second principal components (PCs) of genetic variations. PC1 explained 1.79% of the total variation, and PC2 explained 1.53% of the total variation. The accession IDs are consistent with those in Table 1. The ellipse draws a 95% confidence level with a *t*-distribution.

After SNP sites that were subject to linkage disequilibrium (LD) were pruned, a total of 1673 SNPs were retained. The results of the principal component analysis (PCA) showed that the first and the second PCs (PC1 and PC2) accounted for 1.79% and 1.53% of the total genetic variation, respectively. Along both the PC1 and the PC2 axes, the distribution of *S. acutisepala* was slightly overlapped with that of *S. gemmata* (95% confidence level with a *t*-distribution), showing genetic divergence (Figure 3). Among *S. gemmata* individuals, gem5 (sampled from Mt. Tianmu, Zhejiang) showed the closest genetic affinity with *S. acutisepala* individuals, which is consistent with the results of phylogenetic analysis (Figure 1).

**Figure 4:**
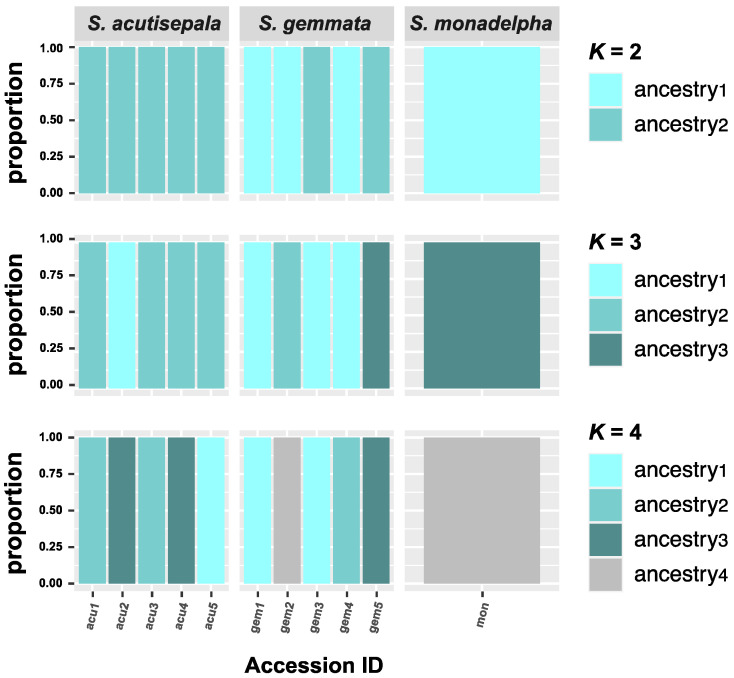
The ADMIXTURE results showing the ancestries of 11 studied individuals through the *K* = 2 to *K* = 4 scenarios. Under each scenario, the individual ancestries of *S. acutisepala* can all be found within *S. gemmata*, and no genetic admixtures were found among individuals (ancestry proportion (*Q*) > 0.99). The accession IDs are consistent with those in Table 1.

The ADMIXTURE analysis suggested a clear and unmixed ancestry for all studied individuals (*Q* > 0.99) (Figure 4). Under the *K* = 2 scenario, *S. gemmata* showed two ancestries; one was shared by *S. monadelpha*, and the other was shared by all *S. acutisepala* individuals. Under the *K* = 3 scenario, all three ancestries were present in *S. gemmata*, two of which were found in *S. acutisepala*. Under the *K* = 4 scenario, a similar pattern also emerged. *S. gemmata* individuals possessed four ancestries, three of which could be found in *S. acutisepala*.

## 3. Discussion

The taxonomic rank of *S. acutisepala* has been discussed for a long time. Some scholars recognized *S. acutisepala* as a different species from *S. gemmata* (formerly *S. sinensis*) [27,28,40], while others argued that *S. acutisepala* was a variety under *S. gemmata* (formerly *S. sinensis*) [21,22]. This dispute is commonplace in taxonomic research since presumed nascent species defined by phenotypic evidence have often been treated as subspecies [41]. More importantly, none of these taxonomic works put emphasis on the genetic differentiation between these two taxa. As supported by our phylogenomic analysis, a progenitor–derivative relationship between *S. gemmata* and *S. acutisepala* is clear (Figure 1) [10]. Compared to other plant species endemic to eastern Asia, the extent of overall genetic divergence between *S. acutisepala* and *S. gemmata* (*F*_st_ = 0.073) is relatively little and comparable to a value among populations rather than between species [42,43]. For example, the inter-lineage *F*_st_ of *Cercidiphyllum japonicum* populations was determined to be 0.07, while that between *Cercidiphyllum japonicum* and its sister species (*Cercidiphyllum magnificum*) was inferred to be 0.72 [44]. Taking the observed genomic divergence and inferred individual ancestry together (Figure 2, Figure 3 and Figure 4), we deduce that *S. acutisepala* is in its infant speciation from *S. gemmata*. Therefore, we recommend treating *S. acutisepala* as a variety of *S. gemmata* as indicated by genetic evidence, which is supported by *FOC* [22].

According to some phylogenetic species concepts, the monophyly of lineages should never be overstated [7]. However, the paraphyly of the progenitor species is not rare in peripatric or budding speciation, or speciation events that follow long-distance dispersals [45,46,47]. Given enough time waiting for the genetic coalescence to happen, it is predictable that gene trees for both *S. acutisepala* and *S. gemmata* will transition from paraphyly to reciprocal monophyly [10,47]. Distinguishable morphological differences have been and are considered important, even essential, in most if not all “scientific” taxonomies as well [48]. In the case of distinguishing *S. acutisepala* from *S. gemmata* in the field, a rough observation of barks may help. The bark exfoliations of *S. acutisepala* are reddish-brown and membranous, while those of *S. gemmata* are yellowish or grayish and hard papery [27,28]. Moreover, we are looking forward to the taxonomic revisions of the *S. gemmata* complex (including *S. gemmata* and *S. acutisepala*) in the upcoming editions of other regional floras by taking the genetic evidence shown here into consideration.

Besides *S. acutisepala*, *S. brevicalyx* was also presumed to be part of the *S. gemmata* complex [25]. Unfortunately, we are not able to include any *S. brevicalyx* accessions in the molecular analysis. According to the literature where the name *S. brevicalyx* was published, the author stated that it is morphologically distinct from *S. gemmata* in having widely ovate to subcordate (vs. ovate) bracts and two nearly orbicular or reniform (vs. ovate) outer sepals [20]. During our field surveys at Mt. Tianmu and its adjacent mountainous areas, where *S. brevicalyx* was reported to be exclusively found, we failed to mark any individuals that perfectly fit the descriptions in the protologue of *S. brevicalyx*. It is most likely that *S. brevicalyx* is conspecific with *S. gemmata* and the type specimen of *S. brevicalyx* only represents an extreme within the phenotypic continuums of *S. gemmata* [28], but still, we encourage a phylogenetic study on historical materials from herbarium specimens [49] to further determine the species entity and the phylogenetic position of *S. brevicalyx*.

Though limited by the type of genomic data, in which only a fraction of the genome was investigated, and the number of studied individuals, the present study provides crucial clues for future studies regarding the speciation mechanisms of *S. acutisepala*. In speciation genomics, genomic regions with higher divergence than expected (i.e., the genomic island of divergence/differentiation) were intensely explored to determine evolutionary events (such as gene flow, modes of selection, the strength of drift, and the formation of loci related to the emergence of reproductive isolation) associated with the speciation process [50,51,52,53], which has been preliminarily revealed here (Figure 2). As the cost of de novo sequencing and assembly of chromosomal-level genomes continues to decrease [54], a reference genome of the *S. gemmata* complex along with whole-genome resequencing data from its natural populations is desperately needed to reveal a comprehensive genomic landscape of *S. acutisepala*, which would undoubtedly facilitate our understanding of the speciation mechanism of this nascent species [55].

## 4. Materials and Methods

### 4.1. Plant Sampling and DNA Extraction

Healthy plant leaves were collected from ten representative individuals of the *S. gemmata* complex (including five of *S. gemmata* and five of *S. acutisepala*) and one individual of *S. monadelpha* as the outgroup (Table 1). The sampling sites of the *S. gemmata* complex can well represent its natural distribution [25]. Specifically, *S. gemmata* accessions were collected from five sites from eastern to southern China, spanning approximately ten degrees of longitude and five degrees of latitude, and *S. acutisepala* accessions were collected from three sites in Zhejiang province, eastern China (Figure 1). Leaf tissue was dried in silica gel for later DNA extraction. Voucher specimens were curated in the Herbarium of Zhejiang University (HZU).

Total genomic DNA was isolated using the Plant DNAzol^TM^ Reagent (Invitrogen, Carlsbad, CA, USA) following the manufacturer’s protocol. The degradation degree of DNA products was assessed using agarose gel electrophoresis, and the concentration of isolated DNA was measured with a Qubit^®^ 3.0 Fluorometer (Thermo Fisher Scientific, Waltham, MA, USA). The DNA products were frozen at −20 °C until use.

### 4.2. RAD-Seq and Data Assembly

Qualified DNA samples (total DNA mass > 300 ng, DNA concentration > 10 ng/μL, and no evident degradation) were delivered to the sequencing institution (Novogene Co., Ltd., Beijing, China) for RAD-seq. DNA samples were fragmented by the *EcoR* I enzyme. DNA fragments of the desired length were gel-purified before adapter ligation and DNA cluster preparation. The detailed method of RAD-seq can be found in the work of Emerson et al. [56]. The RAD-seq libraries were sequenced using the Illumina HiSeq^TM^2000 platform (Illumina, San Diego, CA, USA) to generate 150 bp paired-end reads. After sequencing, raw reads were filtered by removing adapter sequences and low-quality reads (reads with quality lower than 50% or reads in which ambiguous bases made up more than 10% of the raw read). The filtering procedure was performed at the sequencing institution with in-house scripts.

Then, we assembled the clean reads using ipyrad (v. 0.9.90) [57] with default settings. To exclude genetic data from non-nuclear inheritances (e.g., plastome), we used the genome sequence of *Camellia sinensis* [58] as the reference during the data mapping process. The ipyrad pipeline generates a set of outputs for downstream analysis, including a sequence alignment file that contains the full dataset from all assembled loci and a vcf file that stores SNP information. The alignment file would be used in phylogenetic inference, and the vcf file would be used in the analysis of population structure.

### 4.3. Inference of Phylogenetic Tree

Based on the sequence alignment generated above, we reconstructed the consensus ML phylogenetic tree using IQ-TREE (v. 2.2.0.3) with 1000 ultra-fast bootstrap replicates (-B 1000) [59,60]. The best-fit DNA substitution model was determined automatically with ModelFinder as supported by IQ-TREE (-m MFP) [61]. The phylogenetic tree was visualized using tvBOT (v. 2.6) [62] with Bézier curves.

### 4.4. Analysis of Genetic Divergence

Prior to the analysis of genetic divergence, we conducted data filtering on the original vcf file generated from the ipyrad pipeline using vcftools (v. 0.1.16) [63]. We retained SNP loci that met the following criteria: (1) depth ranged from 25% to 75% in depth quartiles (Figure S1); (2) biallelic SNPs; (3) SNPs covered more than 70% of all individuals (--minDP 29 --maxDP 68 --min-alleles 2 --max-alleles 2 --max-missing 0.7). Then, we estimated the genetic divergence between *S. gemmata* and *S. acutisepala* (weighted Weir and Cockerham’s *F*_st_) with vcftools on both the per-site basis (--weir-fst-pop) and the windowed basis (--weir-fst-pop --fst-window-size 100 --fst-window-step 10) using vcftools.

Further, we pruned SNP sites that were subject to high levels of LD using plink (v. 1.90) (--indep-pairwise 10 10 0.4) [64]. Then, we conducted a PCA with plink. Also, we inferred individual ancestries using ADMIXTURE (v. 1.3.0) [65] through the number of clusters (*K*) of two to four. The patterns of genetic divergence within the *S. gemmata* complex were visualized using the R package ggplot2 (v. 3.4.4) [66].

## 5. Conclusions

Based on genome-wide SNPs and a suite of phylogenomic analyses, our results indicate that *S. acutisepala* should be recognized as a variety of *S. gemmata* given that they represent distinct evolutionary lineages and some distinguishable phenotypic differences are already documented. *S. acutisepala* is genetically diverging from *S. gemmata*. Though paraphyly is currently present in *S. gemmata*, both taxa will be monophyletic given adequate time. The present study highlights the power of RAD-seq and SNPs in solving recalcitrant taxonomic issues, especially in the presence of nascent speciation.

## Figures and Tables

**Table 1:** Information on plant accessions used for RAD-seq in this study.

Species	Locality	Voucher No.	Longitude	Latitude	ID	Clean Data (Gb)
*S. acutisepala*	Mt. Tiantai, Tiantai Co., Taizhou, China	H. Lin 16232	121.068863	29.278347	acu1	2.96
*S. acutisepala*	Wuyanling National Nature Reserve, Taishun Co., Wenzhou, China	H. Lin HZU13869	119.669257	27.716245	acu2	4.72
*S. acutisepala*	Mt. Baishanzu, Qingyuan Co., Lishui, China	H. Lin 21013	119.197744	27.762519	acu3	3.95
*S. acutisepala*	Mt. Baishanzu, Qingyuan Co., Lishui, China	H. Lin 21014	119.196900	27.761892	acu4	5.45
*S. acutisepala*	Mt. Baishanzu, Qingyuan Co., Lishui, China	H. Lin 21015	119.197056	27.762336	acu5	4.80
*S. gemmata*	Mt. Tiantangzhai, Jinzhai Co., Lu’an, China	W. Li LWH201704	115.787014	31.135476	gem1	3.39
*S. gemmata*	Mt. Mang, Yizhang Co., Chenzhou, China	H. Lin 16154	112.930632	24.940206	gem2	2.75
*S. gemmata*	Mt. Gutian, Kaihua Co., Quzhou, China	H. Lin HZU13983	118.152874	29.255328	gem3	3.87
*S. gemmata*	Huaping National Nature Reserve, Longsheng Co., Guilin, China	H. Lin 17499	109.929795	25.606376	gem4	3.42
*S. gemmata*	Mt. Tianmu, Linan Co., Hangzhou, China	X. Zheng ZXM00054	119.448134	30.348420	gem5	2.85
*S. monadelpha*	Mt. Ohdai, Yoshino, Nara, Japan	S. Sakaguchi & D. Takahashi SS111-3	135.877671	34.352534	mon	3.09

## Data Availability

The data presented in this study are openly available in GenBank under the BioProject accession number PRJNA1089257.

## Supplementary Materials

The following supporting information can be downloaded at https://www.mdpi.com/article/10.3390/plants13101296/s1: Figure S1: The site depth summary of SNPs generated from the ipyrad pipeline; Table S1: Morphological characteristics, distribution areas, and taxonomic treatments of the *Stewartia gemmata* complex; Table S2: Summary statistics of assembled loci among 11 accessions.
