# Supplementary material for: A Diverging Species within the Stewartia gemmata (Theaceae) Complex Revealed by RAD-Seq Data
Source: Plants (Basel). 2024 May 8;13(10):1296. doi: 10.3390/plants13101296 (PMC11124813; doi:10.3390/plants13101296)
Supplement: Supplementary file 1 [file plants-13-01296-s001.zip › plants-2945321-supplementary.pdf]

Table S1 Morphological characters, distribution areas, and taxonomic treatments of the *Stewartia gemmata* complex

Basionym	Publication	Distribution area	Bark characters	Bracteole and sepal characters	A systematic study on the genera *Stewartia* and *Hartia* (Theaceae) (Li, 1996)	*Flora of China* (Ming and Bartholomew, 2007)	*New Edition of Flora of Zhejiang* (Chen, 2021)
S. gemmata S. S. Chien & W. C. Cheng	Contrib. from Biol. Lab. Sci. Soc. China Bot. Ser. 1931, 6, 66–69.	eastern to southern China (mainly Zhejiang, Anhui, Jiangxi, Fujian, Hunan, Guangxi, and Guangdong)	exfoliations yellowish or grayish and hard papery	Leaflike, ovate, long ovate, or ovate-lanceolate, apex acute to acuminate.	Treated as a synonym of *S. sinensis* var. *sinensis* but was resurrected by Lin et al. (2022).	Followed Li (1996)	Treated as *S. gemmata*.
S. brevicalyx S. Z. Yan	Acta Phytotaxon. Sin. 1981, 19, 462–471.	Mt. Tianmu (northern Zhejiang) and its adjacent areas	exfoliations yellowish or grayish and hard papery	Not leaflike, bracteoles broadly ovate to subcordate and apex obtuse, outer two sepals orbicular or reniform and apex rounded.	Treated as *S. sinensis* var. *brevicalyx*.	Followed Li (1996)	Treated as a synonym of *S. gemmata*.
S. acutisepala P. L. Chiu & G. R. Zhong	Plant Divers. 1988, 10, 1–3.	southern Zhejiang of eastern China	exfoliations reddish-brown and membranous	Leaflike, ovate, long ovate, or ovate-lanceolate, apex acute to acuminate.	Treated as *S. sinensis* var. *acutisepala*.	Followed Li (1996)	Treated as *S. acutisepala*.

Table S2 Summary statistics of assembled loci among 11 accessions

Accession ID	Reads mapped to reference	Total clusters	Loci in assembly
acu1	165851	41360	2515
acu2	637969	90495	9410
acu3	487360	90398	7774
acu4	647343	106318	9045
acu5	598449	101785	8762
gem1	283434	63891	4788
gem2	389016	83033	5542
gem3	463720	83984	7677
gem4	281430	64553	4163
gem5	209144	47039	3201
mon	296416	62561	4537

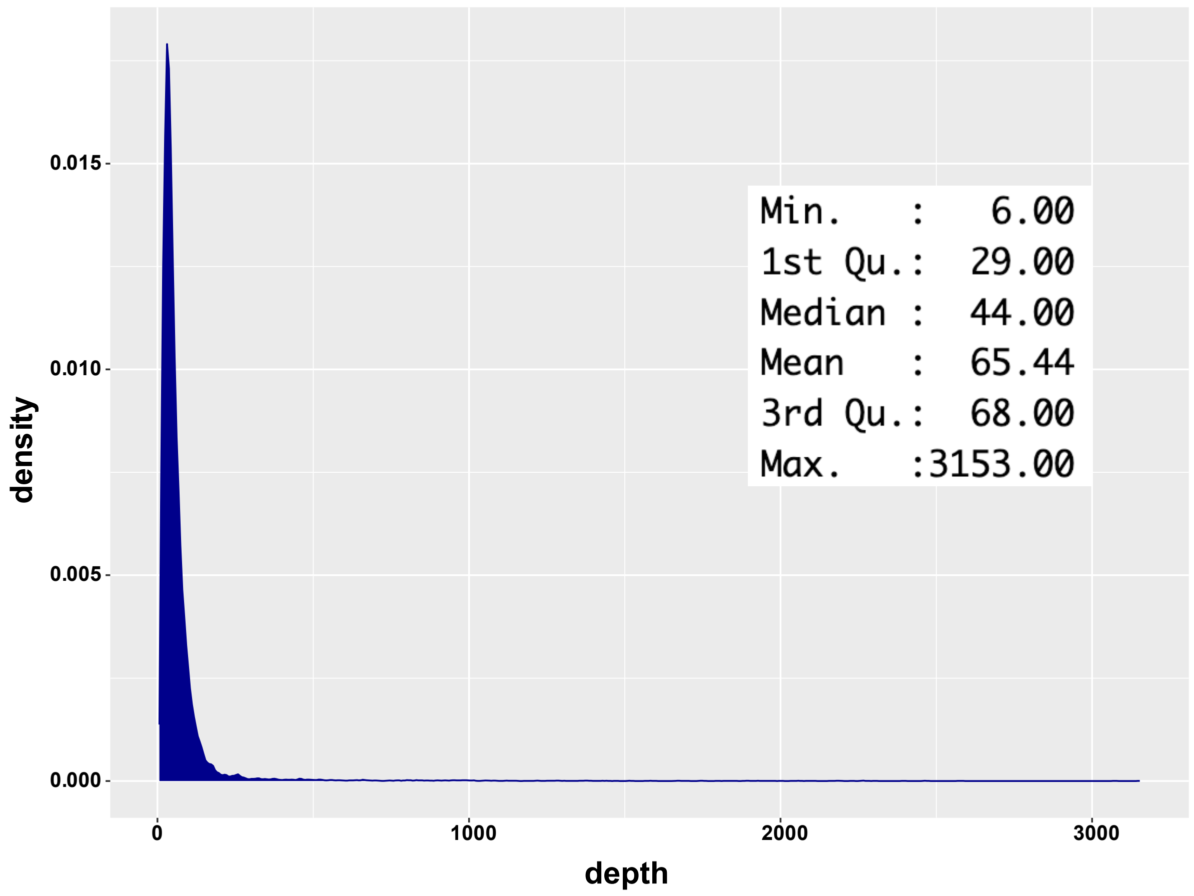

Figure S1 The site depth summary of SNPs generated from the ipyrad pipeline
